# Traumatic brain injury enhances neuroinflammation and lesion volume in caveolin deficient mice

**DOI:** 10.1186/1742-2094-11-39

**Published:** 2014-03-03

**Authors:** Ingrid R Niesman, Jan M Schilling, Lee A Shapiro, Sarah E Kellerhals, Jacqueline A Bonds, Alexander M Kleschevnikov, Weihua Cui, April Voong, Stan Krajewski, Sameh S Ali, David M Roth, Hemal H Patel, Piyush M Patel, Brian P Head

**Affiliations:** 1 Veterans Affairs San Diego Healthcare System, 3350 La Jolla Village Drive, San Diego, CA 92161, USA; 2 Department of Anesthesiology, University of California, San Diego, La Jolla, CA 92093, USA; 3 Neuroscience Research Institute, Scott & White Hospital, Central Texas Veterans Health System, Temple, TX, USA; 4 Department of Surgery, Department of Neurosurgery, Department of Neuroscience and Experimental Therapeutics, College of Medicine, Texas A&M Health Science Center, Temple, TX, USA; 5 Department of Neurosciences, University of California, San Diego, 9500 Gilman Drive, La Jolla, CA 92093, USA; 6 Sanford-Burnham Medical Research Institute, La Jolla, CA, USA; 7 Department of Anesthesiology, Beijing Tiantan Hospital, Capital Medical University, Beijing, China; 8 Center for Aging and Associated Diseases, Helmy Institute of Medical Sciences, Zewail City of Science and Technology, Giza, Egypt

## Abstract

**Background:**

Traumatic brain injury (TBI) enhances pro-inflammatory responses, neuronal loss and long-term behavioral deficits. Caveolins (Cavs) are regulators of neuronal and glial survival signaling. Previously we showed that astrocyte and microglial activation is increased in Cav-1 knock-out (KO) mice and that Cav-1 and Cav-3 modulate microglial morphology. We hypothesized that Cavs may regulate cytokine production after TBI.

**Methods:**

Controlled cortical impact (CCI) model of TBI (3 m/second; 1.0 mm depth; parietal cortex) was performed on wild-type (WT; C57Bl/6), Cav-1 KO, and Cav-3 KO mice. Histology and immunofluorescence microscopy (lesion volume, glia activation), behavioral tests (open field, balance beam, wire grip, T-maze), electrophysiology, electron paramagnetic resonance, membrane fractionation, and multiplex assays were performed. Data were analyzed by unpaired *t* tests or analysis of variance (ANOVA) with *post-hoc* Bonferroni’s multiple comparison.

**Results:**

CCI increased cortical and hippocampal injury and decreased expression of MLR-localized synaptic proteins (24 hours), enhanced NADPH oxidase (Nox) activity (24 hours and 1 week), enhanced polysynaptic responses (1 week), and caused hippocampal-dependent learning deficits (3 months). CCI increased brain lesion volume in both Cav-3 and Cav-1 KO mice after 24 hours (*P* < 0.0001, n = 4; one-way ANOVA). Multiplex array revealed a significant increase in expression of IL-1β, IL-9, IL-10, KC (keratinocyte chemoattractant), and monocyte chemoattractant protein 1 (MCP-1) in ipsilateral hemisphere and IL-9, IL-10, IL-17, and macrophage inflammatory protein 1 alpha (MIP-1α) in contralateral hemisphere of WT mice after 4 hours. CCI increased IL-2, IL-6, KC and MCP-1 in ipsilateral and IL-6, IL-9, IL-17 and KC in contralateral hemispheres in Cav-1 KO and increased all 10 cytokines/chemokines in both hemispheres except for IL-17 (ipsilateral) and MIP-1α (contralateral) in Cav-3 KO (versus WT CCI). Cav-3 KO CCI showed increased IL-1β, IL-9, KC, MCP-1, MIP-1α, and granulocyte-macrophage colony-stimulating factor in ipsilateral and IL-1β, IL-2, IL-9, IL-10, and IL-17 in contralateral hemispheres (*P* = 0.0005, n = 6; two-way ANOVA) compared to Cav-1 KO CCI.

**Conclusion:**

CCI caused astrocyte and microglial activation and hippocampal neuronal injury. Cav-1 and Cav-3 KO exhibited enhanced lesion volume and cytokine/chemokine production after CCI. These findings suggest that Cav isoforms may regulate neuroinflammatory responses and neuroprotection following TBI.

## Background

Traumatic brain injury (TBI) is the leading cause of morbidity and mortality among young people in the Western world. Patients with TBI sustain long-term neurological, cognitive and behavioral deficits leading to a greater requirement for institutional and long-term care. Despite intensive investigative efforts, there is a paucity of interventions designed to reduce morbidity and mortality associated with TBI [1].

Immediately following TBI, there is a substantial excess release of neurotransmitters such as glutamate and signaling nucleotides such as adenosine. Excessive glutamate leads to hyperactivation of N-methyl-D-aspartate receptor (NMDAR) and subsequent excitotoxic neuronal injury. Recent data indicate that hyperactivation of glutamate receptors is short lived (< 1 hour), and there is a substantial reduction in NMDAR expression and signaling within 48 hours of injury [2,3]. Signaling pathways and molecules that are normally associated with neuronal survival (such as BDNF, TrkR, Src, ERK, cAMP and CREB) are reduced for several weeks following TBI [2,4,5]. In addition to glutamate release and neuronal loss, TBI can also produce astro- and microgliosis and enhance the production of proinflammatory cytokines [6-9]. This increased cytokine production can result in alterations in synaptic connections that can lead to additional neuronal loss. The latter effect can contribute to post-traumatic epilepsy (PTE) and long-term behavioral dysfunction with few therapies readily available [10-13].

Membrane/lipid rafts (MLRs) are discrete regions of the plasma membrane enriched in cholesterol, glycosphingolipids and sphingomyelin, and the cholesterol binding and scaffolding protein caveolin (Cav). Three isoforms exist, with Cav-1 and Cav-2 usually co-expressed in a wide variety of tissues, while Cav-3 is canonically expressed in striated muscle [14]. All three isoforms have been described in the central nervous system (CNS) [15-17]. Cav-1 participates in the inflammatory response to the endotoxin lipopolysaccharide through toll-like receptor 4 (TLR4) and through negative regulation of endothelial nitric oxide synthase (eNOS) [18]. Cav-3, normally associated with striated muscles, is not well studied in the CNS. We have recently shown that astrogliosis and microgliosis is increased in the brains of young Cav-1 knock-out (KO) mice [19], and that Cav-1 and Cav-3 modulate microglia morphology [20]. It is therefore conceivable that Cav-1 and Cav-3 might play an important role in the neuroinflammatory response in the brain following controlled cortical impact (CCI). To address this hypothesis, we first performed a variety of assays on wild-type (WT) mice with and without CCI (that is, histological, biochemical, electrophysiological, and by electron paramagnetic resonance (EPR)) to demonstrate establishment of the TBI model. We next conducted CCI and measured the neuroinflammatory response in the brains of WT, Cav-1 KO and Cav-3 KO mice subjected to CCI.

## Materials and methods

### Animal care

All animals were treated in compliance with the Guide for the Care and Use of Laboratory Animals (National Academy of Science, Washington, DC, USA). All animal-use protocols were approved by the Veterans Administration San Diego Healthcare System Institutional Animal Care and Use Committee (IACUC, San Diego, California, USA) prior to performed procedures. C57BL/6 WT and Cav-1 KO mice were purchased from Jackson Laboratories (Bar Harbor, ME, USA) and Cav-3 KO mice were a kind gift from Drs Ishikawa (Professor, Cardiovascular Research Institute, Yokohama City University School of Medicine, Yokohama, Japan) and Hagiwara (Professor, National Institute of Neuroscience, Kodaira, Tokyo, Japan) [21].

### Reagents

The following primary antibodies were used for Western blot (WB) and immunofluorescence microscopy (IF) analysis: Abcam (1 Kendall Square, Suite B2304, Cambridge, MA 02139-1517, USA) - A_2A_AR #ab79714, β_3_-tubulin #ab11314, Cav-3 #ab2912, MAP2 #ab32454; BD Transduction Labs (2350 Qume Drive, San Jose, CA 95131, USA) - NR2B #610417, TrkB #610102; Cell Signaling (3 Trask Lane, Danvers, MA, 01923, USA) - AMPAR #2460 s, Cav-1 #3267, NR1 #4204, NR2A #4205, PSD-95 #2507; Epitomics (863 Mitten Road, Suite 103, Burlingame, CA, 94010-1303, USA) - LDLR #1956-1, LRP-1 #2703-1; Imgenex (11175 Flintkote Ave, Suite E, San Diego, CA, 92121, USA) - GAPDH #IMG-5019A-1; Millipore (290 Concord Road, Billerica, MA, 01821, USA) - GFAP AB5541; Santa Cruz (10410 Finnell Street, Dallas, TX, 75220, USA) - A_1_AR sc-28995, A_3_AR sc-12938, TLR4 sc-30002, goat anti-mouse IgG-HRP sc-2031, goat anti-rabbit IgG-HRP, sc-2030 goat anti-rat IgG-HRP sc-2006; Stressgen (4243 Glanford Avenue, Victoria, BC, Canada) - HSP90 #SPA835; WAKO (1-2 Doshomachi 3-Chome, Chuo-Ku, Osaka, 540-8605, Japan) - Iba1 WB #016-20001, IF #019-19741; Molecular Probes (3175 Staley Road, Grand Island, NY, 14072, USA) - goat anti-rabbit-488 IgG (H + L) #A11008, goat anti-mouse-594 IgG (H + L) #A11005.

### Controlled cortical impact model of traumatic brain injury

CCI was performed as described previously [22]. Briefly, isoflurane (2% vol/vol) anesthetized mice were fixed into a stereotactic frame, maintaining basal temperature (37°C) throughout the procedure. A burr hole was made approximately 5 mm anterior to posterior (0 to −5 A-P) from the bregmatic suture and 4 mm laterally from the sagittal suture over the right hemisphere. Craniotomies were made with a portable drill over the right parietotemporal cortex and the bone flap was removed. Using a stereotaxic impactor (Impact One™; myNeuoLab.com), a 3-mm tip was accelerated down to a 1 mm depth at a speed of 3 m/second with an 85 ms dwell time.

### Histology (n = 4/group) and immunofluorescence (n = 3/group)

For histology, animals were transcardially perfused with 4% paraformaldehyde in 0.1 M PO_4_ buffer then stored in the same buffer for 24 hours and processed for paraffin embedding. Serial sections through the hippocampus (two 5-μm sections per slide, 100 μm apart) were stained with Masson’s trichrome. Digital virtual slides obtained with Aperio Scanscope CS-1 scanner were used for extensive computer assisted morphometry in a Spectrum image analysis system (Aperio Technology Inc., 1700 Leider Lane, Buffalo Grove, IL, 60089, USA). Scanscope software and associated algorithms were applied for measurements of lesion volume and the count of dead or viable neurons in the impact zone, penumbra and relevant area of the contralateral hemisphere control (internal control) as described by Krajewska and colleagues [23]. Whole brains were perfused with 4% paraformaldehyde, cryoprotected with 30% sucrose and frozen for cryostat sectioning in optimal cutting temperature embedding media. Free floating sections (50 μm) were washed in phosphate-buffered saline, blocked and incubated overnight with primary antibodies followed by species-specific secondary antibodies. Species-specific fluroconjugated Alexa® (3175 Staley Road, Grand Island, NY, 14072, USA) secondary antibodies were used at a 1:500 dilution with DAPI in 10% goat blocking solution. Sections were incubated for 1 to 2 hours at room temperature, gently rotating. We have previously characterized and optimized our immunofluorescence protocols for GFAP (glial fibrillary acidic protein), Iba1 (ionized calcium-binding adapter molecule 1) and MAP2 (microtubule associated protein 2) as previously described [19,20,24,25]. Incubation with 10% goat and no primary antibodies, with and without secondary antibodies, served as controls samples for these experiments. Coverslips or brain sections were mounted with an anti-fade solution and imaged; when appropriate, matched exposures were obtained. All other images were exposure and saturation optimized. All quantitation was done using NIH Image J.

### Cognitive and motor tests (n = 20/group)

Male mice (2 to 3 months old) were subjected to CCI and monitored for an additional 3 months followed by a behavioral battery. Open field activity allows assessment of basic activity and general behavior/anxiety of the mouse. Locomotion was recorded and analyzed by a computerized video tracking system (Noldus XT 7.1, 1503 Edwards Ferry Road, Suite 310, Leesburg, VA, 201276, USA). Animals were habituated to the testing room; spontaneous locomotion was assessed in a white plexiglass open field box (41 × 41 × 34 cm enclosures) for 10 minutes. Recorded parameters were distance moved (cm), velocity (cm/second), and time spent in the center of the arena represented by 50% of the total arena (seconds). The wire grip test tests the ability of mice to hang on a metal rail [26]. The metal wire is situated 40 cm from the ground and a soft surface is placed below the wire to prevent physical trauma to the mice. Latency to fall was timed and the test was repeated three times with an inter-trial interval of 30 seconds. The highest latency to fall was multiplied with the body weight to present the holding impulse (seconds × g). In the beam-walking test, mice traverse an elevated narrow beam to reach a platform. The protocol described here measures foot slips while crossing the beam. The apparatus was custom made according to a published protocol of Carter and colleagues [27] with the height of the apparatus set at 50 cm. Continuous alternating T-maze test was used to assess the cognitive ability of the CCI mice; this enclosed apparatus is in the form of a T placed horizontally. Animals are started from the base of the T and allowed to choose one of the goal arms abutting the other end of the stem. Two trials are given in quick succession; on the second trial the rodent tends to choose the arm not visited before, reflecting memory of the first choice, termed as ‘spontaneous alternation’. We assessed this tendency in a test with 14 possible alternations according to plans and a protocol from a previously published method [28,29].

### Electrophysiology (n = 4/group)

Transverse hippocampal slices were prepared as previously described [30]. Mice were anesthetized with isoflurane before decapitation. The brain was quickly removed and immersed for 2 minutes in ice-cold artificial cerebrospinal fluid (ACSF) containing 119 mM NaCl, 2.5 mM KCl, 2.5 mM CaCl_2_, 1.3 mM MgSO_4_, 1 mM NaH_2_PO_4_, 26 mM NaHCO_3_, 10 mM glucose, osmolarity 310 mOsm, continuously bubbled with carbogen (95% O_2_-5% CO_2_), pH 7.4. The hippocampus was extracted and cut in ice cold ACSF with a vibratome (Leica 1000, 1700 Leider Lane, Buffalo Grove, IL, 60089, USA) into 350 μm slices, which were allowed to recover in oxygenated ACSF at 35°C for 30 minutes, and then at room temperature for at least 1 hour prior to experimental recordings.

A slice was transferred into the submerged recording chamber and superfused with ACSF at a constant rate of 1.0 ml/minute at 32°C. To prevent de-oxygenation of ACSF in the recording chamber, the surface was continuously blown over by carbogen warmed to 32°C. Recording electrodes were made of borosilicate glass capillaries (1B150F, World Precision Instruments, Sarasota, FL, USA) and filled with ACSF (resistance 0.3 to 0.5 MΩ). Monopolar stimulating electrodes were made of Pt/Ir wires of diameter 25.4 μm (PTT0110, World Precision Instruments) and had 100 μm long exposed tips. The stimulating and recording electrodes were inserted under visual control perpendicular to the slice surface into the CA1 stratum radiatum 80 to 100 μm from the pyramidal layer, at a distance of 300 to 350 μm apart from each other. The magnitude of monosynaptic responses was evaluated as initial slope of field excitatory postsynaptic potential at latencies 0.1 to 0.9 ms, and the magnitude of polysynaptic responses as the averaged amplitude at latencies 12 to 45 ms after the stimulus. Testing stimuli (duration 100 μs, currents 60 to 80 μA) evoked field responses with amplitudes of 70 to 80% of maximum. Long-term potentiation was induced by tetanizations consisting of a single train of stimuli: 1 second at 100 Hz.

### Superoxide measurements in synaptosomes by electron paramagnetic resonance (n = 3/group)

Brain NADPH oxidase (Nox) activity was assayed by detecting superoxide radical in synaptosomal isolations using EPR spin trapping spectroscopy according to a previously published protocol [31]. Synaptosomal protein (0.2 to 0.5 mg) was mixed with 70 mM 5-(diethylphosphoryl)-5-methyl-1-pyrroline-N-oxide (Axxora, San Diego, CA, USA) and combinations of the substrates/inhibitors was loaded into a 50 μl glass capillary and introduced into the EPR cavity of a MiniScope MS300 Benchtop spectrometer (Louis-Bleriot-Str. 5, D-12487, Berlin, Germany) at a constant temperature of 37°C. Time evolution of the EPR spectra was recorded over 11 minutes from triggering Nox activity by adding appropriate combinations of substrates. For correlative analysis, the signals were quantified over the acquisition time of approximately 6 minutes (that is, the area under oxidative burst curves and normalized by the protein concentration). EPR conditions were as follows: microwave power, 5 mW; modulation amplitude, 2 G; modulation frequency, 100 kHz; MW frequency, 9.49 kHz; sweep width, 150 G centered at 3349.0 G; scan rate, 7.5 G/s and each spectrum was the average of 2 scans.

### Cell culture

Primary cells were isolated using a Papain dissociation kit (#3150; Worthington Chemicals, Lakewood, NJ, USA) as previously described [20,24,25]. Cultures were obtained from post-natal day 3 mouse pups. Mixed glia were separated from neurons according to manufacturer’s instructions and grown to confluence in T-75 flasks in Dulbecco’s modified Eagle’s medium with 10% fetal bovine serum.

### Sucrose density fractionation and Western blot (n = 4/group)

Mouse cortex (50 to 100 mg) was homogenized using a carbonate lysis buffer (500 mM sodium carbonate, pH 11.0) containing protease and phosphatase inhibitors. Lysates were sonicated (three cycles for 15 seconds on ice). Protein was quantified by Bradford assay and normalized to 1 mg/ml. Sucrose was dissolved in MES buffered saline (25 mM MES (2-(*N*-morpholino)ethanesulfonic acid) and 150 mM NaCl, pH 6.5) buffer to prepare 80%, 35% and 5% solutions [25]. Sucrose gradients were prepared by adding 1 ml 80% sucrose followed by 1 ml sonicated sample with brief vortexing followed by 6 ml 35% sucrose followed by 4 ml 5% sucrose. Gradients were spun in an ultracentrifuge using an SW-41 rotor at 39 krpm at 4°C for 3 hours. Fractions (1 ml) were collected from the top of each tube starting at 4 ml to 12 ml. CCI samples were run as individual fractions and f4-6 (buoyant fractions; BF) and f10-12 (heavy fractions; HF) combined for WB. Samples were run on 10% or 4 to 12% bis-tris gels. After transfer to polyvinylidene fluoride membranes, samples were incubated with blocking buffer (3% bovine serum albumin in 20 mM Tris buffered saline containing 1% Tween) for 30 minutes and then incubated overnight with primary antibodies (in blocking buffer) at 4°C. Next day, membranes were washed (3 × 15 minute washes) and re-incubated with species-specific secondary antibodies conjugated to horseradish peroxidase from Santa Cruz at 1:5000 dilution in blocking buffer for 1 hour at room temperature. After extensive washing (4 to 5 × 15 minute washes) membranes were incubated with enhanced chemiluminescence reagent (Amersham Biosciences, PO Box 117, Rockford, IL, 61105, USA) and imaged with the UVP BioSpectrum Imaging System (UVP, 2066 W. 11th Street, Upland, CA, 91786 and saved as .tif files. Densitometric analysis was measured as previously described [25].

### MAGPIX cytokine multiplex assay (n = 6/group)

CCI or sham was performed on the WT, Cav-1 KO and Cav-3 KO mice (2 to 3 months old) and cytokine multiplex assay was performed on the cortex 4 hours post-CCI. Cortices were harvested and frozen 4 hours post-CCI separately from each hemisphere in liquid nitrogen. Frozen tissue was homogenized following the manufacturer’s instructions and 25 μl undiluted homogenate was added to 25 μl assay buffer. Magnetic beads (bead size = 6.45 μm) coated with specific antibodies (RCYTOMAG-80 K-PMX) were added to this solution and the reaction was incubated at 4°C for 24 hours. The beads were washed and incubated with 24 μl biotinylated detection antibody at room temperature for 2 hours. Completing the reaction, 25 μl streptavidin–phycoerythrin conjugate compound was added and allowed to incubate for 30 minutes at room temperature. Beads were washed and incubated with 150 μl sheath fluid for 5 minutes. Concentration of the samples was determined by Bio-Plex Manager version 5.0, after fluorescent capture, and MAGPIX xPONENT software (Millipore, 290 Concord Road, Billerica, MA, 01821, USA [32]. The assays were run in triplicate to confirm the results. Samples were normalized to total protein concentration. Samples were analyzed for the following: IL-1α, IL-1β, IL-2, IL-4, IL-5, IL-6, IL-10, IL-12p70, IL-13, IL-17, IL-18, IFNγ, induced protein 10, chemokine C-C motif ligand (CCL)2 (previously known as monocyte chemoattractant protein 1; MCP-1), CCL3 (previously known as macrophage inflammatory protein 1 alpha; MIP-1α), CCL5 (also known as Regulated upon Activation Normal T-cell Expressed; RANTES), TNFα, vascular endothelial growth factor, eotaxin, growth related oncogene KC (keratinocyte chemoattractant) (CXCL1), leptin, granulocyte colony-stimulating factor, and granulocyte-macrophage colony-stimulating factor (GMCSF).

### Statistical analysis

All data were analyzed by unpaired *t* tests or analysis of variance (ANOVA) with *post-hoc* Bonferroni’s multiple comparison or Student Neuman Keuls test as appropriate. Significance was set at *P* < 0.05. All data are presented as mean ± SEM. All statistical analysis was performed using Prism 6 (GraphPad Software, Inc., 7825 Fay Avenue, Suite 230, La Jolla, CA, 92037.

## Results

### Verification of a controlled cortical impact model of traumatic brain injury shows neuronal damage after 24 hours

**Figure 1:**
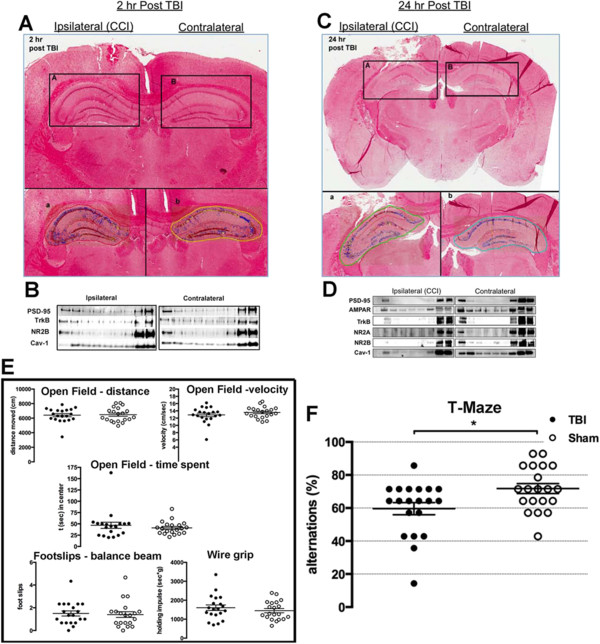
**Controlled cortical impact (CCI) is a viable model of murine traumatic brain injury (TBI). (A)** Trichrome stained paraffin section 2 hours post-CCI with both ipsilateral and contralateral hemispheres. Bottom panels (a) and (b) are the enlargements of the hippocampal area outlined. **(B)** Sucrose density fractionation (SDF) to purify membrane/lipid rafts (MLRs) from ispilateral and contralateral hemispheres. Buoyant fractions contain the cholesterol and sphingolipid enriched MLR, while heavy fractions contain non-MLR cellular components. Western blot of SDF purification of MLR from ipsilateral and contralateral hemispheres 2 hours post-CCI. **(C)** Trichrome stained paraffin section 24 hours post-CCI shows considerable damage to ipsilateral cortex and underlying hippocampus. Bottom panels (a) and (b) are the enlargements of the hippocampal area outlined. **(D)** Western blot of SDF purification of MLR from ipsilateral and contralateral hemispheres 24 hours post-CCI. **(E)** Behavior battery tests performed 3 months post-CCI: open field (distance, velocity, time spent), footslips and wire grip. **(F)** T-maze alternation behavioral test on sham and CCI groups after 3 months.

To assess cortical and hippocampal damage after CCI, serial coronal sections of the brain were prepared and stained with Masson’s trichrome. Figure 1A (2 hours post-CCI) and Figure 1C (24 hours post-CCI) are coronal sections showing cortical lesions. The inserts (boxed areas) are representative of the underlying hippocampal regions (a and b). Neuronal injury was analyzed for dying neurons by Aperio ScanScope imaging and Spectrum analysis algorithm packages as described by Krajewska and colleagues [22], with dead neurons indicated by red/brown coloring superimposed. The results showed minimal hippocampal cell death at 2 hours post-CCI (n = 4) in either ipsilateral or contralateral hemispheres but considerable cell death in CA1 and CA3 is evident at 24 hours post-CCI (n = 4) (Figure 1C-a). Sucrose density fractionation revealed that MLR localization of synaptic proteins and receptors (PSD-95, TrkB, NR2B) and Cav-1 was still intact 2 hours post-CCI (Figure 1B), but there was a drastic reduction after 24 hours (Figure 1D). These results show that there is CA1 neuronal cell death 24 hours post-CCI and a loss in MLR-localized pro-survival and pro-growth synaptic components.

### Hippocampal-dependent learning is decreased 3 months post-controlled cortical impact

Behavioral analysis revealed no significant difference between CCI (n = 20) and sham (n = 20) for open field distance (cm) moved (CCI versus sham: 6,413 ± 217 versus 6,479 ± 216; *P* = 0.793), velocity (cm/second) (CCI versus sham: 12.88 ± 0.49 versus 13.56 ± 0.34; *P* = 0.41) or time spent in the center of the arena (seconds) (CCI versus sham: 46.9 ± 6.8 versus 41.12 ± 3.25; *P* = 0.61) (Figure 1E). Furthermore no significant difference was seen in foot slips on the balance beam (CCI versus sham: 1.5 ± 0.2 versus 1.4 ± 0.3; *P* = 0.56) or the holding impulse (seconds x grams) in the wire grip test (CCI versus sham: 1,606 ± 146 versus 1,450 ± 110; *P* = 0.4). However, a significant difference between groups was recorded in the alternations made (% alternations) in the continuous alternating T-maze (CCI versus sham: 59.6 ± 3.7 versus 71.8 ± 3.0; *P* = 0.028) (Figure 1F). Taken together, the results suggest that no gross difference between groups was present in read outs of basic activity, general behavior/anxiety and neuromuscular function, yet there was a difference in hippocampal dependent ‘spontaneous alternations’, suggesting that the hippocampal injury detected histologically in Figure 1C and the subcellular biochemical changes seen in Figure 1D may contribute to the hippocampal-dependent behavioral changes.

### Controlled cortical impact model of traumatic brain injury enhances polysynaptic responses in isolated hippocampal slices at 1 week

**Figure 2:**
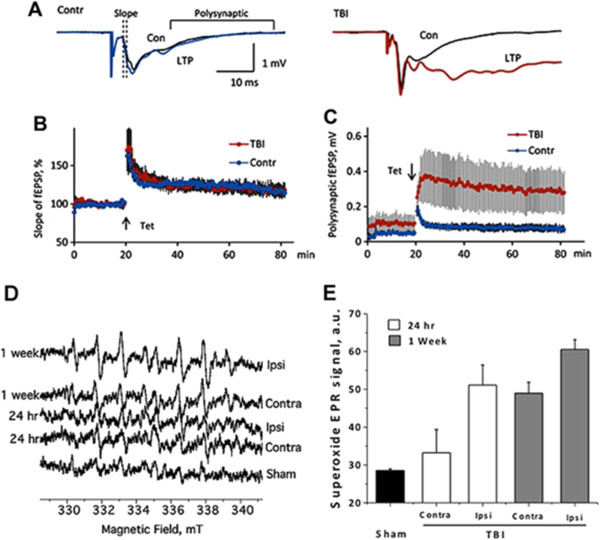
**Electrophysiological properties of the commissural-collateral input in the CA1 region of the hippocampus at 1 week post-traumatic brain injury (TBI). (A)** Representative responses in slices from the contralateral (Contra; left) and the TBI (controlled cortical impact (CCI), right) hippocampus before and after the tetanus. **(B)** Long-term potentiation (LTP) of monosynaptic responses after CCI. **(C)** Changes of polysynaptic response (averaged amplitude at latencies 12 to 45 ms after the stimulus). **(D)** Enhanced NADPH oxidase (Nox) activity in both ipsilateral and contralateral hemispheres 24 hours and 1 week post-TBI as shown by increased superoxide electron paramagnetic resonance (EPR) signal amplitude relative to sham animals. **(E)** Isolated synaptosomes from the ipsilateral side exhibited greater Nox activity, which increased 1 week post-CCI. Con, control; Contra, contralateral; fEPSP, field excitatory postsynaptic potential; Ispi, ipsilateral.

Electrophysiological changes were assessed in hippocampal slices isolated from contralateral (n = 4) and ipsilateral (n = 4) hemispheres. No changes in long-term potentiation of monosynaptic responses were observed (Figure 2A,B). However, changes in the response shape were more pronounced in the ipsilateral (CCI) versus the contralateral slices. Thus, the averaged amplitude of the field potentials at 14 to 45 ms that represent mostly polysynaptic responses were considerably greater in the ipsilateral versus contralateral slices from CCI brains (Figure 2A,C). The observed increase in polysynaptic responses in the CCI hippocampal hemisphere is an indicator of increased pro-epileptic activity, and this neurophysiological change could be an important component that contributed to the behavioral change observed in Figure 1F.

### Controlled cortical impact model of traumatic brain injury exhibits enhanced NADPH oxidase activity

To test if injury-induced neuronal loss and the subsequent neuroinflammation in our current TBI model are associated with Nox activation, Nox activity was assessed 24 hours (n = 3) and 1 week (n = 3) post-CCI by EPR (Figure 2D,E). TBI mice exhibited enhanced Nox-derived superoxide generation 24 hours and 1 week after CCI in both contralateral and ipsilateral hemispheres. Interestingly, increased Nox activity in the contralateral side indicates that ‘global’ brain inflammation was induced 1 week post-CCI.

### Caveolin knock-out animals have altered expression of membrane/lipid raft localized neuronal and glial proteins

**Figure 3:**
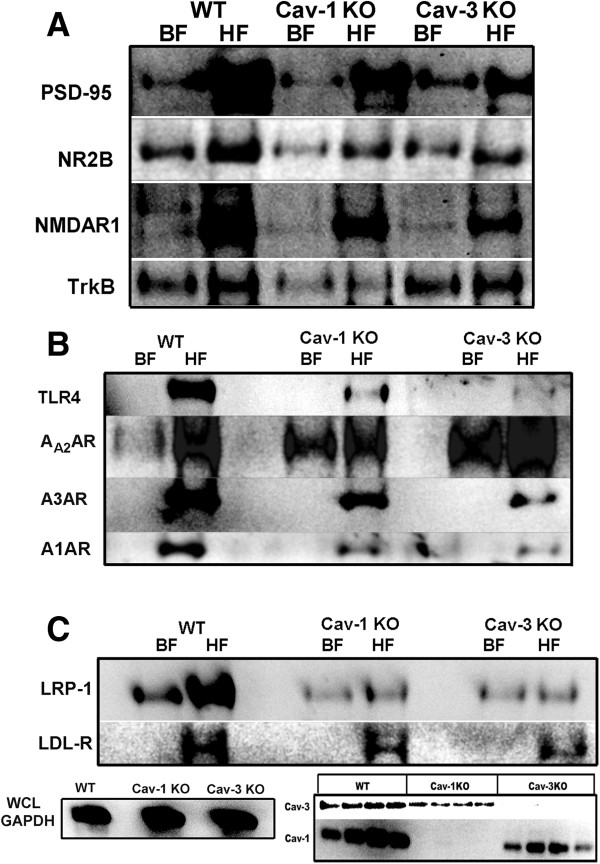
**Caveolin (Cav)-1 knock-out (KO) and Cav-3 KO mice have altered expression and membrane/lipid raft (MLR) localization of key neuronal and glial proteins.** Sucrose density fractionation (SDF) and Western blot (WB) of wild-type (WT), Cav-1 KO, and Cav-3 KO brains. Buoyant fractions (BF) contain the cholesterol and sphingolipid enriched MLR, while heavy farctions (HF) contain non-MLR cellular components. **(A)** WB detection of PSD-95, NR2B, NR1 and TrkB expression in BF and HF. **(B)** WB detection of toll-like receptor-4 (TLR4), A_A2_AR, A_3_AR, and A_1_AR expression in BF and HF. **(C)** WB detection of LRP-1 and LDL-R expression in BF and HF. Bottom left, WB analysis of GAPDH in whole cell lysates (WCL) from which SDF were generated for each group. Bottom right, WB shows loss of Cav-1 and Cav-3 protein expression in the transgenic mouse used in the present study.

WT, Cav-1 KO and Cav-3 KO mouse cortex were homogenized and processed for sucrose density fractionation to analyze neuronal and glial proteins (Figure 3). BF (consisting of fractions 4 to 6) and HF (consisting of fractions 10 to 12) were used for WB. PSD-95, NR2A, NR2B, and TrkB were all reduced in both BF and HF from Cav-1 KO brains, results akin to our previously published work [19]. BF from Cav-3 KO brains showed increased expression of PSD-95, NR2B, NR1A, and TrkB (Figure 3A) compared to Cav-1 KO, yet the pattern-recognition receptor TLR4 was nearly lost in HF from Cav-3 KO brains and decreased in Cav-1 KO (Figure 3B).

Adenosine receptors exhibited differential expression patterns among the three groups (Figure 3B). WT brains showed limited BF localization of A_2A_R, an inflammation promoting subtype of adenosine receptors. Interestingly, A_2A_R expression was enhanced in both Cav KO brains, with Cav-3 KO displaying the highest BF-localized expression. The anti-inflammatory A_1_AR and A_3_AR isoforms were only detected in HF for all groups. Cav-1 and Cav-3 KO mice expressed less A_1_AR and A_3_AR compared to WT.

Because Cavs are cholesterol binding proteins, and lipoprotein receptors LRP-1 and LDL-R subcellularly localize to MLR [33,34], we assessed the expression of these receptors (Figure 3C). Both Cav-1 KO and Cav-3 KO showed decreased expression of LRP-1, with the same ratio of BF to HF. There was little detection of LDL-R in BF from WT, Cav-1 and Cav-3 KO, yet Cav-3 KO showed the least expression compared to Cav-1 KO and WT. The KO phenotype was confirmed by WB for Cav-1 KO and Cav-3 KO hippocampi.

### Caveolin knock-out animals have altered resident central nervous system cell populations

**Figure 4:**
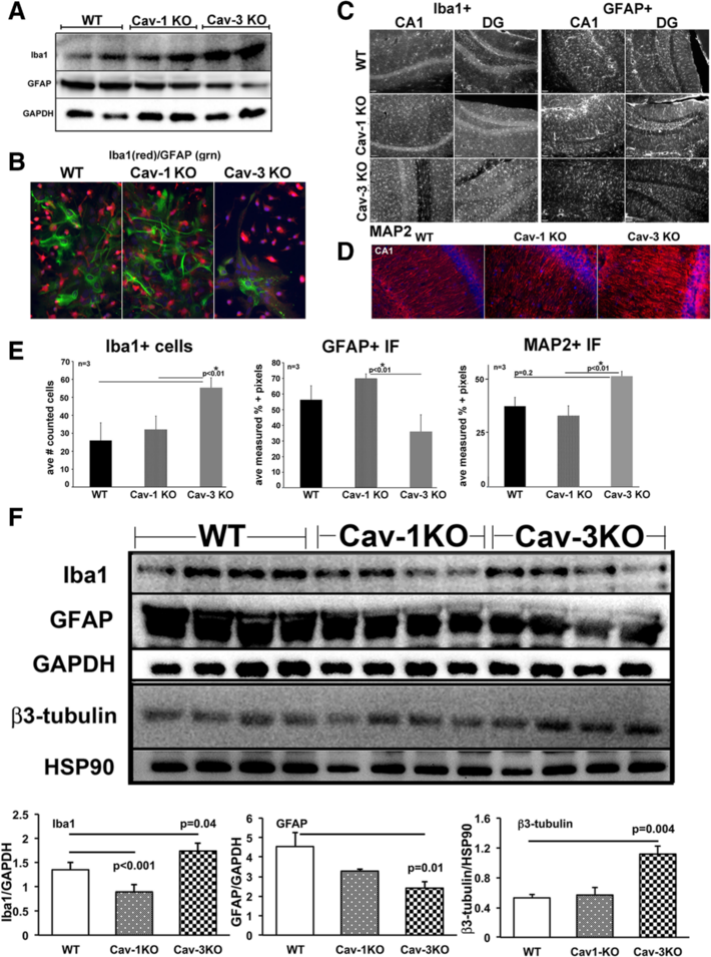
**Caveolin** (**Cav)-1 knock-out (KO) and Cav-3 KO mice have different microglial and astrocyte populations.** Primary mixed glia were cultured from brains from wild-type (WT), Cav-1 KO and Cav-3 KO postnatal day 3 pups. **(A)** Western blot (WB) analysis of GFAP (glial fibrillary acidic protein) and Iba1 (ionized calcium-binding adapter molecule 1) in primary mixed glia cultures normalized to GAPDH. **(B)** Immunofluorescence microscopy of GFAP (green) and Iba1 (red) in primary mixed glia cultures. Nuclei were stained with DAPI. **(C)** Sections of hippocampal CA1 and dentate gyrus (DG) regions from WT, Cav-1 KO and Cav-3 KO mice labeled with Iba1 (left) and GFAP (right). **(D)** Sections of hippocampal CA1 region with the neuronal dendritic marker MAP2 (microtubule associated protein 2). **(E)** Quantitation of cell numbers from n = 3 animals. A statistically significant increase in Iba1 cells is found in Cav-3 KO mice compared to WT (*P* < 0.01, left graph). A significant decrease in GFAP labeling is found in Cav-3 KO mice compared to Cav-1 KO (*P* < 0.01, middle graph), and a trending decrease when compared to WT (not significant). A statistically significant increase in MAP2 labeling is also detected in Cav-3 KO versus Cav-1 KO (*P* < 0.01, right graph). Data displayed as mean ± SEM. **(F)** Bottom panels are quantitative WB analysis of Iba1, GFAP and β_3_-tubulin from mouse hippocampi. Statistically significant increased expression of Iba1 and β_3_-tubulin and decreased GFAP expression was detected in Cav-3 KO mice. Conversely, decreased Iba1 expression was observed in Cav-1 KO.

Primary mixed glial cultures were isolated from WT, Cav-1 KO and Cav-3 KO on postnatal day 3 to match passage and days *in vitro*. WB analysis (Figure 4A) and IF (immunofluorescence microscopy) (Figure 4B) indicate that Cav-1 KO and Cav-3 KO have increased number of Iba1 positive cells and decreased GFAP positive cells compared to WT, with Cav-3 KO cells showing the greatest reduction in GFAP positive cells as indicated by IF (Figure 4B, right image). To confirm these findings, age-matched hippocampi were examined by IF for Iba1 (microglia), GFAP (astrocytes) (Figure 4C) and MAP2 (neuronal dendrites) (Figure 4D). Cav-1 KO brains exhibit slightly increased Iba1 positive microglia and GFAP positive staining in CA1 and dentate gyrus (DG) compared to WT, similar to previously reported findings from our group [19]. Hippocampi from Cav-3 KO brains displayed less GFAP positive cells in the CA1 and DG compared to both WT and Cav-1 KO. WT MAP2 labeling of CA1 pyramidal neurons shows a typical pattern of normally arranged neuronal cell layer and aligned processes in the molecular layer of the DG. Cav-1 KO showed less MAP2 positive neurites, which is consistent with our previous findings [19], yet Cav-3 KO exhibited greater MAP2 staining compared to Cav-1 KO and WT (Figure 4D). Quantitation of the IF images are shown in Figure 4E: Cav-3 KO showed increased Iba1 positive cells (*P* < 0.01, n = 3) compared to WT or Cav-1, less GFAP positive cells (*P* < 0.01, n = 3) compared to WT and Cav-1 KO, and increased MAP2 positive neurites (*P* < 0.01, n = 3) compared to WT and Cav-1 KO. Basal hippocampi from WT, Cav-1 and Cav-3 KO were analyzed by WB for Iba1, GFAP and neuronal specific β_3_-tubulin to assess the cell-specific protein expression pattern (Figure 4F). Iba1 was significantly reduced (*P* < 0.001) in Cav-1 KO and significantly elevated in Cav-3 KO (*P* = 0.04). GFAP was significantly reduced (*P* = 0.01) and β_3_-tubulin was elevated (*P* = 0.004) in Cav-3 KO, findings consistent with IF data.

### Caveolin-1 knock-out and Caveolin-3 knock-out mice exhibit larger lesion volume 24 hours post-controlled cortical impact compared to wild-type mice

**Figure 5:**
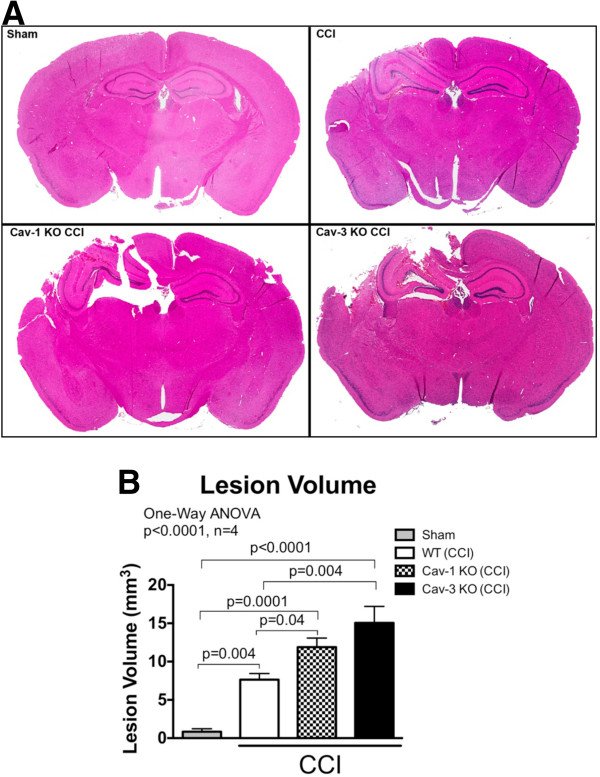
**Controlled cortical impact (CCI) causes a significant larger brain lesion volume in caveolin (Cav)-1 and Cav-3 knock-out (KO) mice compared wild-type (WT).** WT, Cav-1 and Cav-3 KO mice were subjected to CCI and lesion volume was quantitated on Masson’s trichrome stained histological sections 24 hours following impact as previously described [22]. Cav-1 (11.9 ± 1.2 mm^3^) and Cav-3 KO (15.1 ± 2.2 mm^3^) mice had a significant larger brain lesion volume compared to WT (7.5 ± 0.8 mm^3^) and sham (0.8 ± 0.4 mm^3^) (*P* < 0.0001, n = 4). Data displayed as mean ± SEM. **(A)** Representative Masson’s trichrome stained coronal brain sections. **(B)** Quantitation of lesion volume shown in **(A)**.

To assess brain injury after CCI, serial coronal sections of the brain were prepared and stained with Masson’s trichrome and lesion volume was quantitated as previously described [22]. Both Cav-1 (n = 4; 11.9 ± 1.2 mm^3^) and Cav-3 KO (n = 4; 15.1 ± 2.2 mm^3^) had a significantly larger lesion volume compared WT (n = 4; 7.5 ± 0.8 mm^3^) and sham (n = 4; 0.8 ± 0.4 mm^3^) (*P* < 0.0001, Figure 5) 24 hours post-CCI.

### Controlled cortical impact enhances pro-inflammatory cytokines and chemokines in caveolin-1 knock-out and caveolin-3 knock-out mice at 4 hours post-impact

Brain homogenates from WT, Cav-1 KO and Cav-3 KO mice were analyzed for 23 different cytokine/chemokines to assess the inflammatory response in our CCI model. Of the 23 analytes, 10 exhibited significantly different expression patterns among the three groups (Table 1). Sham data from all three groups (WT, Cav-1 KO and Cav-3 KO; n = 6/group) revealed no significant difference in the analytes measured. Of note, many of the measured cytokines from sham (n = 6) samples were below the level of detection, in contrast to the CCI (n = 6) samples which all yielded measurable amounts. Following CCI, both ipsilateral and contralateral hemispheres from Cav-1 (n = 6) and Cav-3 KO (n = 6) exhibited significant elevation in IL-1β, IL-2, IL-6, IL-9, IL-10, IL-17, KC, MCP-1, MIP-1α, and GMCSF compared to the respective transgenic sham corresponding hemispheres (that is, ipsilateral and contralateral) (*P* = 0.0005, two-way ANOVA, Table 1). For WT alone, CCI significantly enhanced IL-1β, IL-9, IL-10, KC, and MCP-1 in ipsilateral hemisphere and IL-9, IL-10, IL-17, and MIP-1α in contralateral versus corresponding sham hemisphere. For Cav-1 KO, CCI significantly elevated IL-1β, IL-2, IL-6, IL-9, IL-10, IL-17, KC, and MCP-1 versus ipsilateral Cav-1 KO sham and significantly increased all 10 cytokines/chemokines versus contralateral Cav-1 KO sham. When compared to WT CCI corresponding hemisphere, Cav-1 CCI increased IL-2, IL-6, KC and MCP-1 in ipsilateral and IL-6, IL-9, IL-17 and KC in contralateral. For Cav-3 KO, all 10 cytokines/chemokines were significantly elevated in both ipsilateral and contralateral versus Cav-3 KO corresponding sham hemispheres. When compared to WT CCI, Cav-3 CCI displayed a significant increase in all 10 cytokines/chemokines in both hemispheres except for IL-17 (ipsilateral) and MIP-1α (contralateral). When compared to Cav-1 KO CCI, Cav-3 KO CCI had a significant increase in IL-1β, IL-9, KC, MCP-1, MIP-1α, and GMCSF in the ipsilateral hemisphere and IL-1β, IL-2, IL-9, IL-10, and IL-17 in the contralateral hemisphere.

**Table 1:** Multiplex array reveals brain changes in certain cytokines, chemokines, and growth factors after CCI

**Ipsilateral**						**Contralateral**						
	**WT**		**Cav-I KO**		**Cav-3 KO**		**WT**		**Cav-1 KO**		**Cav-3 KO**	
	**Sham**	**TBI**	**Sham**	**TBI**	**Sham**	**TBI**	**Sham**	**TBI**	**Sham**	**TBI**	**Sham**	**TBI**
IL-1β	0.072 **±** 0.004	0.83 ± 0.06*	0.06 **±** 0.005	1.4 ± 0.3*	0.07 **±** 0.007	2.5 ± 0.4*#	0.08 **±** 0.01	0.59 **±** 0.07	0.06 **±** 0.005	1.05 ± 0.13*	0.07 **±** 0.01	2.3 ± 0.2*#^
IL-2	011 **±** 0.002	0.36 **±** 0.03	0.11 **±** 0.005	0.7 ± 0.07*#	013 **±** 0.004	**0.96 ± 0.15#**	0.11 **±** 0.01	0.22 **±** 0.05	0.12 **±** 0.004	0.6 ± 0.1*	0.13 **±** 0.01	0.9 ± 0.1*#^
IL-6	0.04 **±** 0.006	2.0 **±** 0.4	0.03 **±** 0.007	30.9 ± 3.9*#	0.03 **±** 0.003	42.2 ± 7.1*#	0.03 **±** 0.005	0.58 **±** 0.06	0.02 **±** 0.001	14.2 ± 3.9*#δ	0.02 **±** 0.005	7.5 ± 1.3*#
IL-9	0.69 **±** 0.03	9.6 ± 0.4*	0.6 **±** 0.06	15.1 ± 1.5*	0.8 **±** 0.09	23.3 ± 3.3*#^	0.79 **±** 0.19	7.4 ± 1.3*	0.8 **±** 0.03	13.6 ± 2.2*#	0.7 **±** 0.06	21.0 ± 1.5*#^
IL-10	0.034 **±** 0.003	1.0 ± 0.08*	0.04 **±** 0.003	1.5 ± 0.2*	0.06 **±** 0.002	2.0 ± 0.3*#	0.046 **±** 0.012	0.62 ± 0.08*	0.04 **±** 0.001	1.03 ± 0.17*	0.05 **±** 0.01	1.8 ± 0.1*#^
IL-I7	0.0009 **±** 0.0003	0.22 **±** 0.02	0.002 **±** 0.0003	0.3 ± 0.04*	0.003 **±** 0.0001	0.4 ± 0.1*	0.0014 **±** 0.0005	0.13 ± 0.03*	0.001 **±** 0.0002	0.3 ± 0.05*#	0.003 **±** 0.001	0.4 ± 0.04*#^
KC	0054 **± 0.**01	6.3 ± 1.2*	0.05 **± 0.**006	31.8 ± 3.1*#	0.05 **±** 0.005	49.1 ± 7.7*#^	0.027 ± 0.009	1.6 **±** 0.2	0.02 **±** 0.001	9.5 ± 1.8*#	0.03 **±** 0.01	11.0 ± 2.5*#
MCP-1	0.051 **±** 0.004	7.0 ± 2.2*	0.05 **±** 0.009	19.4 ± 1.5*#	0.05 **±** 0.004	39.4 ± 6.3*#^	0.042 **±** 0.009	1.9 **±** 0.2	0.04 **±** 0.004	5.6 ± 1.2*	0.07 **±** 0.01	8.6 ± 1.9*#
MIP-1α	0.031 **±** 0.004	2.0 **±** 0.07	0.02 **±** 0.008	6.7 **±** 0.6	0.03 **±** 0.003	20.1 ± 4.8*#^	0.022 ± 0.006	1.6 ± 0.2*	0.01 ± 0.002	2.0 ± 0.6*	0.02 ± 0.01	2.4 ± 0.3*
GMCSF	NA	0.84 **±** 0.2	NA	1.9 **±** 0.6	0.2 **±** 0.03	3.4 ± 0.6*#^	0.14 **±** 0.05	0.7 **±** 0.2	0.11 **±** 0.01	2.0 ± 0.4*#	0.3 **±** 0.04	2.5 ± 0.3*#

Four hours post-CCI, contralateral and ipsilateral hemispheres were analyzed with the MAGPIX Cytokine Multiplex Assay for IL-1β, IL-2, IL-6, IL-9, IL-10, IL-17, KC (keratinocyte chemoattractant), monocyte chemoattractant protein 1 (MCP-1), macrophage inflammatory protein 1 alpha (MIP-1α), and granulocyte-macrophage colony-stimulating factor (GMCSF). Data (n = 6 mice/group) represent mean ± SEM. *P* < 0.05 or less were considered statistically significant. *P < 0.05 versus sham hemisphere, #*P* < 0.05 versus wild-type (WT) traumatic brain injury (TBI) hemisphere, ^*P* < 0.05 versus caveolin (Cav)-1 knock-out (KO) TBI hemisphere, δ*P* < 0.05 versus Cav-3 KO TBI hemisphere. NA, not available (below detection).

## Discussion

The current findings are the first to demonstrate that loss of Cav isoforms produces isoform-specific effects on inflammation in a CCI model of TBI. The objective of the present study was to quantitatively assess neuroinflammation in the brain of Cav-1 KO and Cav-3 KO mice early after CCI. Many previously published studies have evaluated downstream signaling proteins involved in the induction of cytokines/chemokines after injury [35,36], but none have directly investigated the role of Cav and MLR-localized receptors and associated downstream signaling mediators on TBI-induced inflammatory responses. The loss of Cav-1, specifically, has been found to result in increased ischemic damage following transient middle cerebral artery occusion [37]. One possible mechanism for increased injury is a lack of eNOS inhibition by Cav-1 leading to increased metalloproteinase activity and blood–brain barrier degradation [38]. Because both microglia and astrocytes express Cav-1 and Cav-3, it is critical to understand how these proteins regulate receptor signaling, and secondary messengers such as NO, to induce or repress inflammation following CNS injury. Moreover, Cav-1 KO mice have previously been shown to exhibit enhanced anxiety and impaired spatial memory, demonstrating an important role for Cav-1 in normal neurological phenotype [39]. Although it has yet to be determined which cell type contributes to these behavioral abnormalities, our previous work that demonstrates a reduction in MLR and MLR-localized synaptic proteins accompanied with reduced hippocampal synapses does indicate in part that loss of Cav-1 causes cellular morphological changes essential for normal brain physiology regardless of the cell type [19].

Using a well-characterized CCI model of TBI, we detected glial reactivity in the ipsilateral hemisphere 4 hours post-injury and hippocampal neuronal death 24 hours post-injury. Behavioral studies revealed cognitive deficits in working memory, as determined by T-maze, 3 months post-injury with no motor deficits. Not surprisingly, the damage was not limited to the hippocampus, as extensive parietal cortical damage was also evident by 4 hours, which included enhanced neuroinflammation as indicated by the significantly elevated cytokine production in the ipsilateral cortex.

TBI can produce epileptogenesis, a neuropathological change that is frequently associated with depression, anxiety disorders and side effects from anti-epileptic treatments [40]. PTE is a significant complication for the returning Veteran population with estimates that approximately 34% of returning Veterans who experienced moderate to severe head trauma are at risk for developing PTE. The findings from the current study show an increase in polysynaptic responses in the CCI hippocampal hemisphere, an indicator of increased pro-epileptic activity. Such a finding is a potential indicator of increased pro-epileptic activity because aberrant circuit formation is believed to be involved in epileptogenesis [41,42]. Therefore, these results (that is, enhanced polysynaptic responses) could be an important factor contributing to the post-TBI death of hippocampal neurons and development of epilepsy.

Another putative mechanism involved in the development of PTE is enhanced generation of reactive oxygen species [43], as seen in the current study (Figure 2D,E). Previous studies have shown Nox activation leads to increased neurotoxic activation of microglia [44]. Gene array studies have shown that changes in synaptic plasticity, glial proliferation and inflammatory reactivity occur before initial seizures manifest [45,46]. Anti-epileptic drugs, as a prophylactic intervention administered soon after TBI, have shown some efficacy in preventing early seizures (< 1 week), but are ineffective in preventing later, more devastating episodes of seizures [47]. Therefore, more efficacious interventions that attenuate these initial key changes may alter the course of PTE development and potentially reverse the long-term cognitive changes that result from TBI.

We have previously shown a role for Cavs as regulators of neuronal survival [19,24,25] and microglia activation [20]. In an attempt to understand the potential role of Cavs in mediating the early inflammatory responses after TBI, 23 cytokines were measured 4 hours post-injury. Interestingly, 10 analytes were significantly elevated in both hemispheres of brains from either Cav-1 KO or Cav-3 KO mice. Common pro-inflammatory cytokines/chemokines, including IL-1β, IL-2, IL-6, IL-9, IL-10, IL-17, KC, MCP-1, MIP-1α, and GMCSF were upregulated in both Cav KO mice, yet only IL-6, KC, MCP-1, and MIP-1α were significantly elevated with CCI compared to the contralateral Cav. MCP-1 (CCL2) was significantly increased in the contralateral and ipsilateral hemisphere of both Cav KO mice; these results are in agreement with previously published work that demonstrated increased expression in a pilocarpine model of status epilepticus [48]. Persistently elevated expression of MCP-1 in both Cav KO mice indicates a disruption in the normal signaling and trafficking of the MCP-1/CCR2 (MCP-1 receptor) complex, an interesting finding considering that previous work showed that MCP-1 KO mice have attenuated lesion size and less astrogliosis following TBI [49]. Other studies have shown that Cav-1 plays a prominent role in astrocytic responses to MCP-1 by mediation of cellular signaling transduction through caveolae-localized CCR2 [50,51]. Therefore, interventions that increase Cav expression and restore normal CCR2 expression and function may be a potential therapeutic target. As a final Cav-mediated chemokine example from the multiplex analysis, MIP-1α (CCL3), a ligand for CCR5 (MIP-1α receptor), is significantly elevated after CCI in the ipsilateral hemisphere. Although many groups have found increased expression of MIP-1α following induced status epilepticus models, the role for MIP-1α, either protective or inflammatory, is still under debate [52].

Various G-protein coupled receptors that are regulated by Cav, such as adenosine receptors, are involved in the complex process of microglia or astrocyte activation [53-56]. The data from the current study demonstrated reduced expression of adenosine A_1_AR and the anti-inflammatory A_3_AR in both Cav-1 KO and Cav-3 KO brains. Evidence exists that the loss of A_1_AR (A_1_AR KO mice) results in an increased risk for epileptogenesis [57,58]. Because the current data show a reduction in A_1_AR expression in Cav KO mice, loss of Cav isoforms due to injury (as shown in Figure 1D) may render the brain more susceptible to physiological changes (Figure 2C) and subsequent seizure development.

A_2A_AR sits at the intersection of multiple control points for the development of neuropathology and neuropsychiatric conditions (reviewed in [59,60]). Activation of A_2A_AR can negatively affect the functionality of A_1_AR [61], resulting in an enhanced inflammatory state. Additional evidence suggests that A_2A_AR activation plays a major regulatory role in microglia-dependent neurotrophin release and subsequent microglia proliferation during neuroinflammation [62]. The present findings demonstrate that both Cav KO mice have increased MLR localization of the pro-inflammatory A_2A_AR compared to WT (Figure 3B). After injury, local adenosine concentrations greatly increase activating plasmalemmal localized A_2A_AR receptors in microglia [7,63]. The present finding that Cav KO mice exhibit increased MLR-localized A_2A_AR basally may in part explain the elevated cytokine/chemokine production in the brains of these mice both with and without CCI.

Cholesterol is a key component of MLR and for maintaining synaptic integrity. Because synaptic loss is one of the dynamic changes associated with the latency period for development of PTE [64-67], changes in cholesterol homeostasis and MLR integrity may in part contribute to the etiology of PTE. Lipoprotein receptors are key players in cholesterol homeostasis [68], and two important lipoprotein receptors in the brain, LRP-1 and LDL-R, are subcellularly localized to MLR [33,34]. Because Cav KO mice have reduced expression of LRP-1 and to a lesser extent LDL-R compared to WT (Figure 3C), events that cause decreased Cav expression in the brain (age or injury) may reduce cholesterol transport from glia to neurons and therefore increase the risk for synaptic loss, intercellular events we are presently investigating [19].

## Conclusions

We have demonstrated for the first time that loss of Cav isoforms results in enhanced cytokine/chemokine production following TBI. The present study extends previously published results showing the neuropathology of Cav-1 KO mice, and shows for the first time that loss of Cav-3 significantly enhances cytokine/chemokine production in the setting of TBI. The extent of injury and inflammation was considerably greater in the Cav-1 KO and Cav-3 KO mice. Some degree of inflammation is clearly necessary for neuroregeneration and brain repair after TBI. Modulation of the inflammatory response, rather than its suppression, may be necessary. To that end, our data are consistent with the premise that modulation of Cav-1 and Cav-3 levels in a cell-type-specific manner (neurons, astrocytes and microglia) might afford novel therapeutic options for the treatment of TBI.

## Abbreviations

ACSF: artificial cerebrospinal fluid; ANOVA: analysis of variance; BF: buoyant fractions; Cav: caveolin; CCI: controlled cortical impact; CCL: chemokine C-C motif ligand; CNS: central nervous system; DG: dentate gyrus; DMEM: Dulbecco’s modified Eagle’s medium; eNOS: endothelial nitric oxide synthase; EPR: electron paramagnetic resonance; GFAP: (glial fibrillary acidic protein); GMCSF: granulocyte-macrophage colony-stimulating factor; HF: heavy fractions; Iba1: ionized calcium-binding adapter molecule 1; IF: immunofluorescence microscopy; IFN: interferon; IL: interleukin; KC: keratinocyte chemoattractant; KO: knock-out; MAP2: microtubule associated protein; MCP-1: monocyte chemoattractant protein 1; MIP-1α: macrophage inflammatory protein 1 alpha; MLR: membrane/lipid raft; NMDAR: N-methyl-D-aspartate receptor; Nox: NADPH oxidase; PTE: post-traumatic epilepsy; TBI: traumatic brain injury; TNF: tumor necrosis factor; TLR4: toll-like receptor 4; WB: Western blot; WT: wild-type.

## Competing interests

The authors declare that they have no competing interests.

## Authors’ contributions

IRN performed cell culture, biochemistry experiments (WB and IF), and participated in the draft of the manuscript. JMS assisted in behavioral analysis and participated in the draft of the manuscript. LAS performed cytokine array and assisted in analysis. SK and JAB performed CCI experiments. AK conducted electrophysiology experiments. WC assisted in CCI experiments and histology. AV performed behavioral studies. JAB and SK assisted in establishment of CCI model. SSA conducted EPR experiments and analysis. DMR participated in draft of the manuscript. HHP participated in the draft of the manuscript. PMP participated in establishment of CCI model and the draft of the manuscript. BPH participated in establishment of CCI model, study design, data analysis, and draft of the manuscript. All authors read and approved the final manuscript.

## Authors’ information

Work in the authors’ laboratories is supported by Veteran Affairs Merit Award from the Department of Veterans Affairs BX001225 (B. P. Head), BX000783 (D. M. Roth), and BX001963 (H. H. Patel), National Institutes of Health, Bethesda, MD, U.S.A., NS073653 (B. P. Head) and HL091071 and HL107200 (H. H. Patel), Department of Defense W81XWH-10-0847 (S. Krajewski).
